# The Cyrenaica Coastal Survey Project: Documenting Endangered Maritime Heritage in Libya

**DOI:** 10.1007/s11457-022-09336-1

**Published:** 2022-09-13

**Authors:** Julia Nikolaus, Mohamed O. M. Abdrbba, Ahmad Emrage

**Affiliations:** 1 Ulster University, Coleraine, UK; 2 Omar Al-Mukhtar University, Al Bayda, Libya; 3 Benghazi University, Benghazi, Libya

**Keywords:** Libya, Coastal survey, Cyrenaica, Heritage management

## Abstract

This paper introduces the Cyrenaica Coastal Survey (CCS), a collaborative project between the Maritime Endangered Archaeology project and the Department of Antiquities (DoA) Cyrenaica in partnership with the Universities of Al Bayda and Benghazi in Libya. Since the Arab Spring in 2011 and the subsequent civil unrest in Libya, heritage professionals, the DoA, and various individuals interested in heritage have struggled to safeguard heritage sites across the country, as policies and laws that protected archaeological sites were no longer reinforced and adhered to in the wake of the revolution. This lack of finances, capacity, and governmental support led to an unprecedented loss of archaeological sites since 2011. The CCS survey records the current condition of maritime sites along the Cyrenaican coast. The project focuses on the smaller, lesser known, coastal heritage sites that are not as well studied as the much larger classical period port towns of Apollonia, Tocra, or Ptolemais. This article will focus on the results of the first phase of the project between ancient Phycus (modern Zawiet el-Hamama) and Kainopolis (modern Al-Ogla). The results of the first stage of the Cyrenaica Coastal Survey provides a snapshot of the damages and threats that coastal heritage faces in Libya, most notably (often unregulated) building activities, clearance, sand mining, and coastal erosion. Furthermore, this article highlights the importance of remote collaboration between UK institutions, in-country partners, and heritage authorities, especially in countries where the discipline of maritime archaeology has been established more recently.

## Introduction

The province of Cyrenaica is located in eastern Libya, stretching from the Sirte Basin in the west to the Egyptian border in the east. For thousands of years, this coastline has been occupied by humans who made use of the rich marine environment, the fertile coastal plains, and the uplands of the Djebel Akhdar. This long history is best exemplified by evidence from the Haua Fteah cave that was occupied by humans as far back as 150,000–200,000 years ago, where shell middens, fish, and crab remains show that people made use of the resources provided by the nearby sea (Douka et al. 2014). Over the millennia the peoples who lived along the coast of Cyrenaica have left some remarkable evidence of their occupation, most visibly from the Hellenistic to early Islamic periods, when Cyrenaica was closely linked to the rest of the Mediterranean world by seafaring trade. Ancient writers mentioned over 25 sites along the coastline, although for some their exact location remains uncertain (Hesein 2015). The five largest ports in classical antiquity include Euesperides (which was later moved to Berenice), Taucheria, Ptolemais, and Apollonia (e.g., Jones and Little 1971; Bennett et al. 2004; Bogacki 2012; Hesein 2015; Yorke and Davidson 2017). Smaller harbors, settlements, forts, farms, and industrial installation related to maritime and agricultural activities are scattered along the coast and shipwrecks found, for instance, at Apollonia and Ptolemais, speak of the strong connection this region once had with the wider Mediterranean world (Laronde 1990; Tusa 2010; Beltrame 2012; Hesein 2014, 2015; Buzaian 2019; Tusa and Buccellato 2019).

Despite the region's long standing and strong connection to the sea, maritime archaeology remains a peripheral, but growing, branch of archaeology in Libya. A small number of submerged sites have been explored and documented predominantly by foreign missions, including Apollonia, Tocra, and Ptolemais (Flemming 1964, 1971; Jones and Little 1971; Laronde 1990; Tusa 2010; Tsimplis et al. 2011; Pizzinato and Beltrame 2012; Tusa and Buccellato 2019). Libya ratified the UNESCO Convention on the Protection of the Underwater Cultural Heritage in 2005. Sadly, efforts in capacity building and the establishment of a dedicated maritime archaeology unit were hampered by the civil unrest following the Arab Spring in 2011. A maritime archaeology unit was finally established in 2017, but as mentioned above, capacity and funds remain low.

Over the last decades, coastal heritage sites have suffered increasingly from a variety of damages as a result of humans and natural causes. One of the main drivers is the ongoing rapid expansion of settlement along the coastline. In Libya, urban centers are predominantly located in the narrow coastal strip that borders the Mediterranean Sea; over 80% of the country’s population now reside there (Abubrig 2016). This is exacerbated by a sharp rise in population over the last 70 years, from 1,245,358 people in 1950 to 6,871,292 in 2020 (United Nations 2018). Subsequently the coastal zone has undergone large environmental and socio-economic changes in recent decades. Settlements along the coast have grown exponentially, often at the cost of the surrounding natural and historic environment (Bennett and Barker 2011).

This problem has intensified in the aftermath of the Arab Spring of 2011 and the subsequent socio-political unrest. New private, unregulated housing developments have increased dramatically, in part due to a lack in official governance and policy making (Abdulkariem and Bennett 2014; Bennett and Graham 2015; Fitzgerald et al. 2015; Nebbia et al. 2016; Menozzi et al. 2019). Since 2011, it is not only the larger cities and towns that are expanding, but also smaller villages and settlements are growing, and new housing and road developments spring up in areas that were previously uninhabited. The increasing desirability of living along the coast is causing land prices to rise which, in turn, encourages landowners to sell to developers that build domestic houses, holiday homes, and resorts. Agricultural expansion into areas that were previously respected due to the presence of cultural heritage is also an ongoing problem (Abdulkariem and Bennett 2014; Hesein 2015).

The increase in coastal erosion caused by sea level rise and progressively severe winter storms, likely exacerbated by climate change, add to the threats and damages that sites along the Cyrenaican coastline face (Dasgupta et al. 2009). Parts of the coastline are characterized by limestone cliffs that are intersected by deep wadis. Other areas consist of sandy and soft sediments than can be easily washed away by wave action (Hamza et al. 2011; Said 2011; Riad 2016; Catani et al. 2020). The devastating effects are already documented at Tocra and Apollonia (Yorke 1972; Bennett et al. 2004; Bennett 2018). A study by Westley et al. (2021) indicates that already 8–14% of coastal sites in Eastern Libya have been affected by the erosion of the seashore and modelling predicts an increase to 25–26% by 2050, rising to 32–33% by 2100. The high rate of illegal sand mining further increases the speed and level of coastal erosion. Elgazali et al. found that Botrabah beach, east of Tocra, is in critical condition due to high sand removal rates that negatively impact the surrounding environment, including the archaeology, resulting in the erosion of up to 15,680.00 metric tons of soil during the winter storm season (Elgazali et al. 2021). Little is yet known about the impact of industrial waste and seawater pollution on the archaeology as studies on the quality of sea water in Libya are still rare (see, e.g., Bonsignore et al. 2018).

To monitor and protect the numerous sites that are under threat is an almost impossible task for the heritage professionals of the Department of Antiquities (DoA), whose efforts are hindered by low capacity and a lack of funds. Remote sensing studies reveal that many sites across Libya have already been lost to the factors mentioned above and, along with them, the knowledge we could gain about the Libyan past (Nebbia et al. 2016; Rayne et al. 2017a, b, 2020; Tapete and Cigna 2018).

The Cyrenaica Coastal Survey (CCS hereafter) was born out of the imminent and increasing threats that face maritime archaeology in Libya today. This paper presents the results of the first phase of the survey between Kainopolis (modern Al-Ogla) and Phycus (modern Zawiet el-Hamama) giving an overview of the damages and threats these sites face. Two case studies will offer a detailed assessment of the current state of maritime heritage in Libya, followed by a discussion on present and potential future protection, management strategies, collaborations, and capacity building.

## Aims and Methodology

**Fig. 1:**
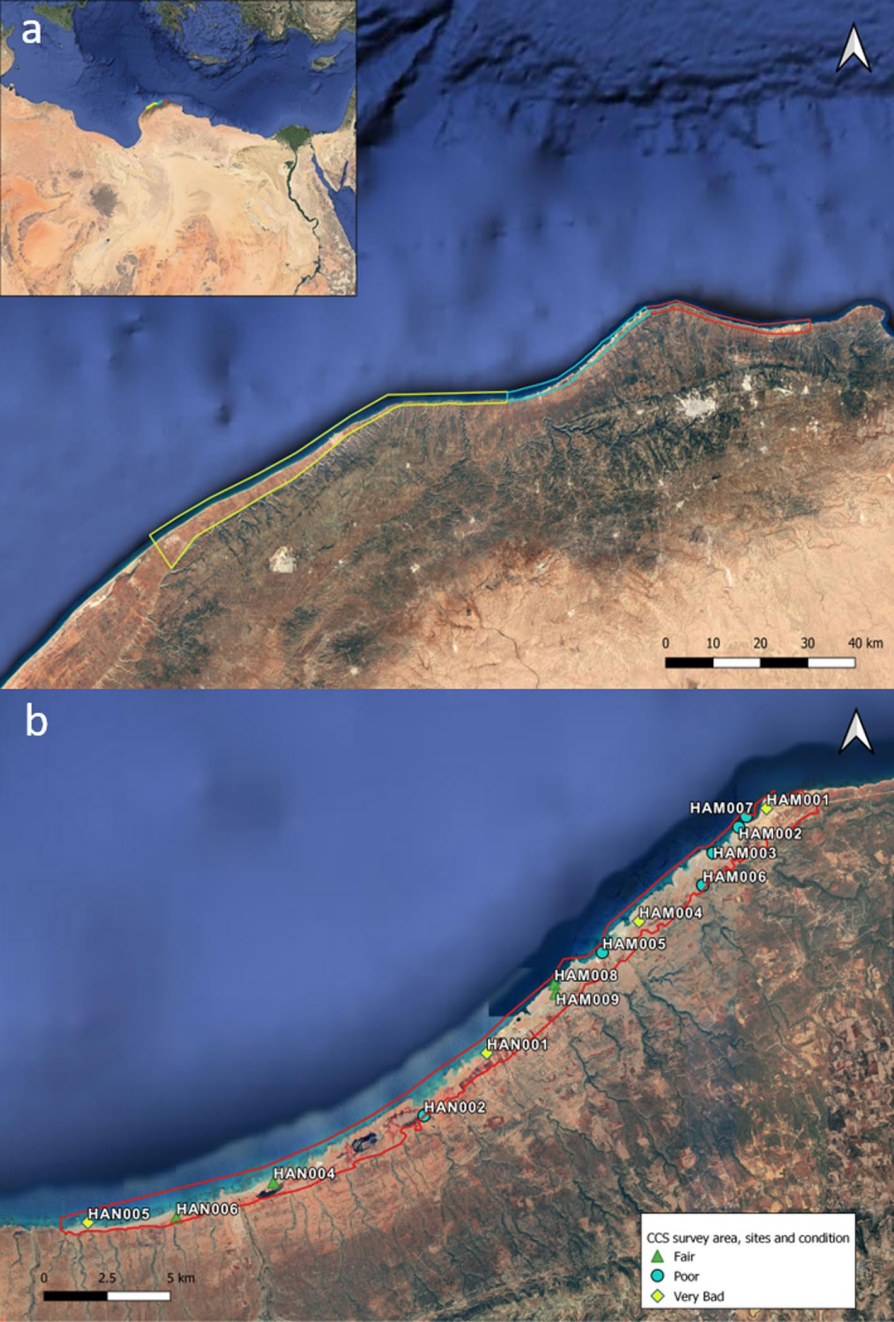
**a** Outline of survey area along the Cyrenaican coastline for phase 1 (blue), phase 2 (red), and phase 3 (yellow). **b** Location and overall conditions of sites surveyed during phase 1 of the project (Basemap: ©Digital Globe via Google Earth)

The CCS project is a collaboration between the Maritime Endangered Archaeology Project (MarEA, based at Ulster University and University of Southampton) and the DoA Cyrenaica in partnership with the universities of Al Bayda and Benghazi. The survey area stretches from Tocra (ancient Tauchaira) to Sousa (ancient Apollonia). The survey was carried out in three stages: stage one between Kainopolis and Phycus in December 2020, stage two was between Apollonia and Phycus in April 2021, and finally stage three between Tocra and Kainopolis in September and December 2021 (Fig. 1a). This contribution will focus on stage one of the CCS project between Kainopolis and Phycus (Fig. 1b).

The CCS survey focused in particular on smaller or lesser-known sites within the study area. Almost no intensive fieldwork has been conducted on these smaller sites, and we still know relatively little about what role these places played within local and wider economic and socio-political networks. Among the most comprehensive works are surveys carried out by Hesein (2014, 2015) and Tusa (2010; Tusa and Buccellato 2019). Hesein focused on recording the smaller harbor sites between Kainopolis and Noat to investigate their local and wider significance within the Mediterranean network. Tusa concentrated on the area between Kainopolis and Apollonia and the area around Derna, where he and his team recorded a wide variety of coastal heritage, including a number of settlements, tombs, harbors, and production sites as well as submerged features and shipwrecks. Other relevant work includes a survey carried out by Emrage (2015) in the region of Wadi al-Kuf in Cyrenaica recording and analyzing the large fortified buildings (gasr) which included the coastal zone between Kainopolis and Phycus. Olive presses and olive oil production facilities at rural sites in the area were surveyed, meticulously recorded, and analyzed by Buzaian (2019). While threats and damages to some sites are pointed out in these previous studies, they are mostly observations that are not analyzed or quantified. Therefore, a systemic condition assessment of sites along the Cyrenaican coastline was still lacking.

The CCS was established to fill this gap in documentation and to add an additional dimension to survey work that had already been undertaken. Instead of recording the archaeological features in detail, the primary aim was to assess and document the current condition of, and damages or threats to, onshore, intertidal, and submerged sites. This documentation is intended to facilitate the development of comprehensive protection and mitigation plans in the future which, ultimately, can help policy makers address challenges to maritime cultural heritage in the country.

The MarEA team carried out detailed remote sensing assessments in preparation of the survey predominantly using open-source satellite imagery available through Google Earth Pro. Very high resolution SkySat satellite imagery from November 2020 was analyzed to identify potential sites, together with relevant published resources and maps. Declassified analog satellite images from the Gambid Keyhole 7 (1966) and Hexagon Keyhole 9 (1974) reconnaissance missions available at a meter to sub-meter resolution were used to observe changes in the coastal landscape, to record the condition of sites over time, and to document sites that are no longer visible on modern imagery.

The survey took place over a period of 10 days with a team of heritage professionals from the DoA, the University of Benghazi and the Omar Al-Mukhtar University, Al Bayda. A diving team from the maritime archaeology unit joined the survey for several days to explore submerged, near-shore features. Sites were recorded with handheld GPS devices and photographs. Dedicated survey forms that mirror the terminology of the EAMENA/MarEA database were translated into Arabic and used by the survey team. The recording forms focus on the documentation and assessment of threats and damages, current condition, and current or foreseen threats to sites. The data recorded during the survey were entered into the MarEA/EAMENA database and are also available to members of the DoA.

## Overview of Disturbances and Threats

**Fig. 2:**
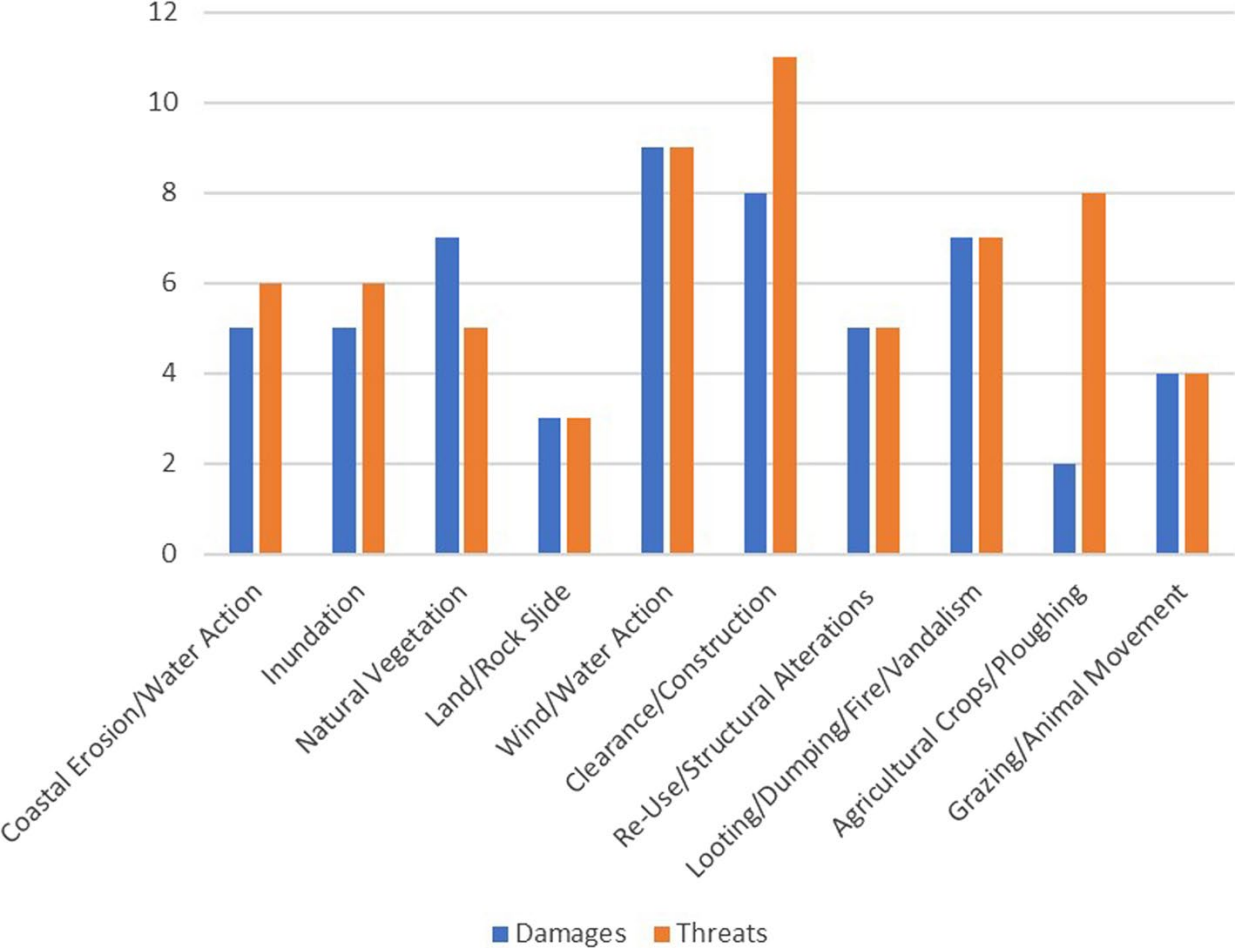
Main types of damage and threats to sites as recorded by the CCS team. A potential rise in clearance and construction, as well as agricultural activities in the future, has been noted

During the survey, the team visited 15 potential sites identified from satellite imagery and from published materials (Fig. 1b). Fourteen locations were confirmed to be of archaeological interest, and 66 sub-sites were recorded, ranging from port facilities and settlements, fortified buildings, farms, quarries, and industrial installations, to graves and rock-cut tombs. The underwater team was hindered by bad weather conditions but was, nevertheless, able to survey three submerged sites (HAM002, HAM007 and HAN001). While none of the main sites were completely destroyed, four were found to be in very bad condition (serious signs of active deterioration and/or signs of severe structural instability), six in poor condition (moderate signs of active deterioration and/or signs of moderate structural instability), and four in fair condition (little evidence of active deterioration or some feature; Fig. 1b). The predominant type of damage that has been recorded can be grouped into the following categories: coastal erosion/water action/recession of water, wind/water action, natural vegetation, land/rockslide, clearance/construction, reuse/structural alteration, agricultural crops/ploughing and vandalism in the form of looting, rubbish dumping, graffiti painting, or small fires (Fig. 2).

### Natural Factors

Most sites recorded are exposed to the elements throughout the year. Over the centuries, wind and rain caused serious erosion, collapse, and structural damage. Soil erosion is a common problem on sites located near the sea as the soil is very friable and washes away easily. Further signs of serious erosion can be seen on building blocks made of the local limestone of which many appear to be porous, and the edges of dressed blocks are rounded from weathering. A scientific study undertaken at the classical period port city of Leptis Magna in western Libya indicates that the levels of carbonic acid in rainwater have a negative impact on the level of erosion of the local limestone (Abd El-Tawab Bader 2014). This would affect sites built of limestone across Libya, including sites in the CCS survey area.

**Fig. 3:**
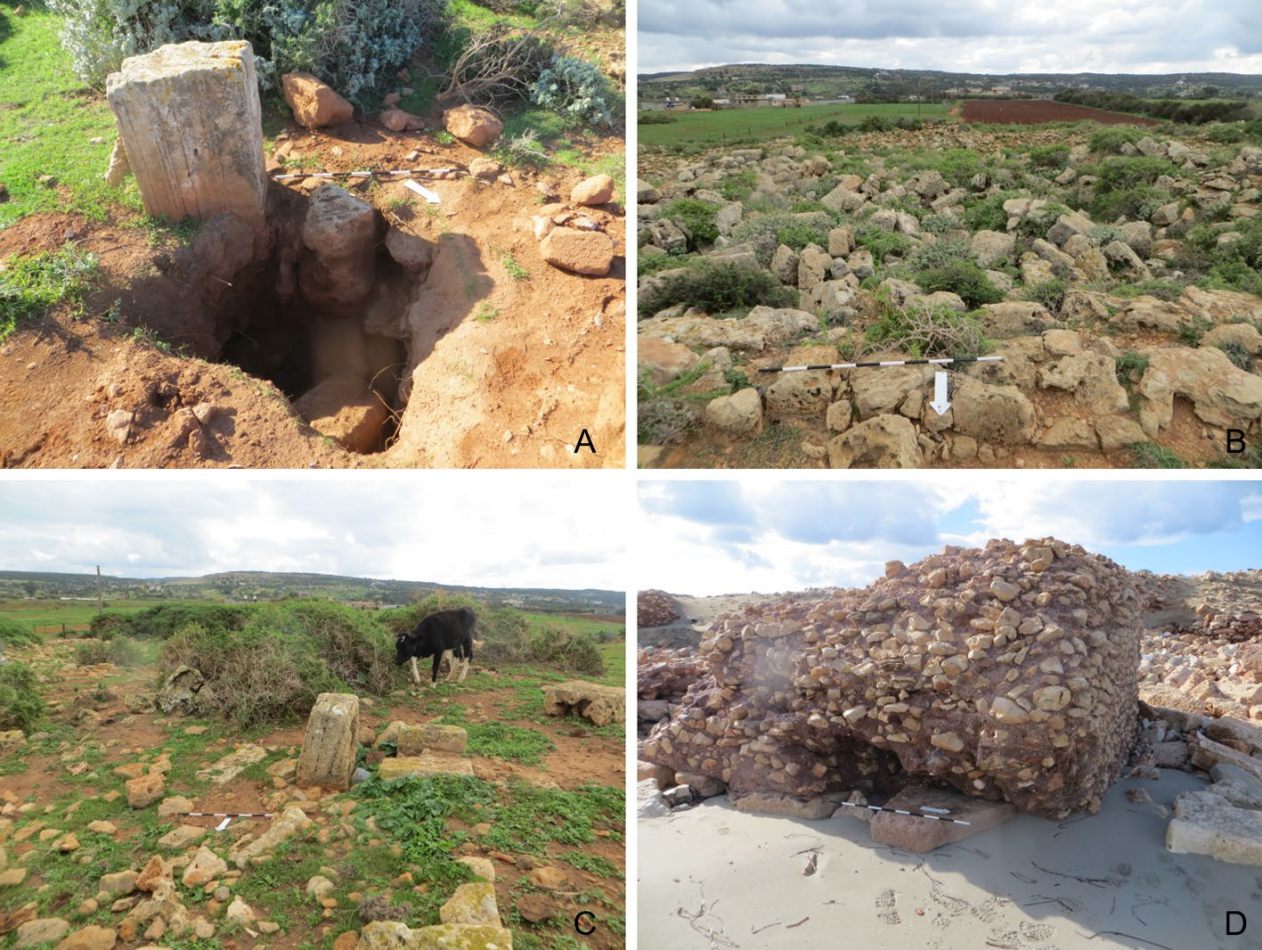
**a** A hole probably dug by looters at HAM009. **b** The remains of the substantial building at HAM005, shrubbery is growing across the site and the stones show signs of weathering. Agricultural fields encroaching the site can be seen in the background. **c** Cows grazing at HAM005. **d** A substantial building at HAN005 that shows signs of erosion caused by wave action

Natural vegetation such as low shrubbery, bushes, and trees cover seven of the 14 sites (Fig. 3b, c). The vegetation increases in density further away from the seashore. While some of the roots might cause damage to existing walls and compromise the integrity of the structure, the vegetation can also protect the limestone structures from the impacts of rain and soil erosion.

Damage directly related to the maritime environment include coastal erosion and retreat, wind and wave action, and recession of water. All sites that are located at the shoreline are impacted by those damages. Erosion, collapse, structural damage and, in some cases, rock and landslides are the most common effects. Like water and wind action further inland, this type of damage is often a slow process. Limestone features such as buildings or rock-cut tanks and vats that are located by the sea are exposed to the corrosive action of saltwater on limestone, which causes it to erode or dissolve at a faster pace (Abd El-Tawab Bader 2014). Recent studies have shown an alarming increase in erosion along the eastern Libyan coast over the last two decades, most likely exacerbated by climate change. For instance, between 2002 and 2019 at Tocra the coastline eroded by approximately 11 m (m) along the central part of the site. Future projections are even more alarming, indicating that by 2050 25–26% of sites will experience the impact of erosion (Westley et al. 2021; Bennett et al. 2004). The results of this survey show that some of the coastal sites are already adversely impacted. This is particularly apparent at HAM001 and HAN001 which are discussed in more detail in the case studies below. High levels of erosion on archaeological features located on the beach can be seen at other sites such as Al-Ogla (HAM005). Here, the sapping of the underlying sand by the undertow undermined the foundations of a large building, possibly a tower, which is causing the structure to tilt and to become increasingly unstable (Fig. 3d). From the limited underwater exploration conducted during the survey, it appears that inundated features predominantly suffer from erosion caused by wave and water action.

### Anthropogenic Impacts

Damage to sites caused by human intervention is closely related to the expansion of settlements and agricultural activities in the area. Eight sites out of 14 were damaged by clearance related to the construction of access roads, houses or, in the case of HAN001, the extension of an existing cemetery located over parts of the ancient settlement. Sites such as HAN005 and HAN006, which are located further away from the main road that connects Zawiet el-Hamama and Zawiet el-Hanya to the large cities of Al Merj and Al Bayada, are not yet affected by clearance and construction. However, the rapidly increasing construction of new access roads and tracks from 2013 onwards indicates that housing developments are slowly creeping closer to sites that were previously protected from such threats due to their remote location.

Two sites were impacted by agricultural development. At HAM005 some of the surrounding area was cleared for the expansion of agricultural fields, impacting the southern, eastern, and western edges of the site. HAM005 was first recorded by Hesein (2015) between 2011 and 2013 and has not yet been studied in detail. It consists of a series of buildings overlooking the sea, wells, cisterns, and quarries. Any evidence for activity along the beach just below the site has been eliminated by sand mining. Hesein (2015, p 455) has not found any industrial features near the site but suggests they are located to the southeast and southwest. Unfortunately, this area is now covered by agricultural fields, and locating evidence for any industrial activities will be very challenging (Fig. 3b). Animal movement and grazing of sheep, goats, and cows are common, especially near settlements (Fig. 3c). On the one hand, some of the natural vegetation is kept at bay. On the other hand, animal hooves can dislodge already loose stones and cause some damage to the archaeology.

Seven of the sites surveyed suffered from some form of vandalism, including looting, littering, small fires, and graffiti. However, looting attempts appear to be sporadic and haphazard, with a relatively small number of looting pits evident, such as at HAN005, HAN006, or at HAM009 (Fig. 3a). A fair amount of litter covering some of the features can be observed particularly around coastal sites, which may partly be the result of rubbish being washed up on the shore. Further damage is caused by the reuse of archaeological features, particularly for storage and animal shelter. Some rock-cut tombs are closed off by doors that were installed much more recently. It is notable that reuse of ancient features occurs primarily around the expanding modern settlements of Zawiet el-Hamama and Zawiet el-Hanya.

## Case Studies

### Phycus (HAM001, HAM002, HAM007)

**Fig. 4:**
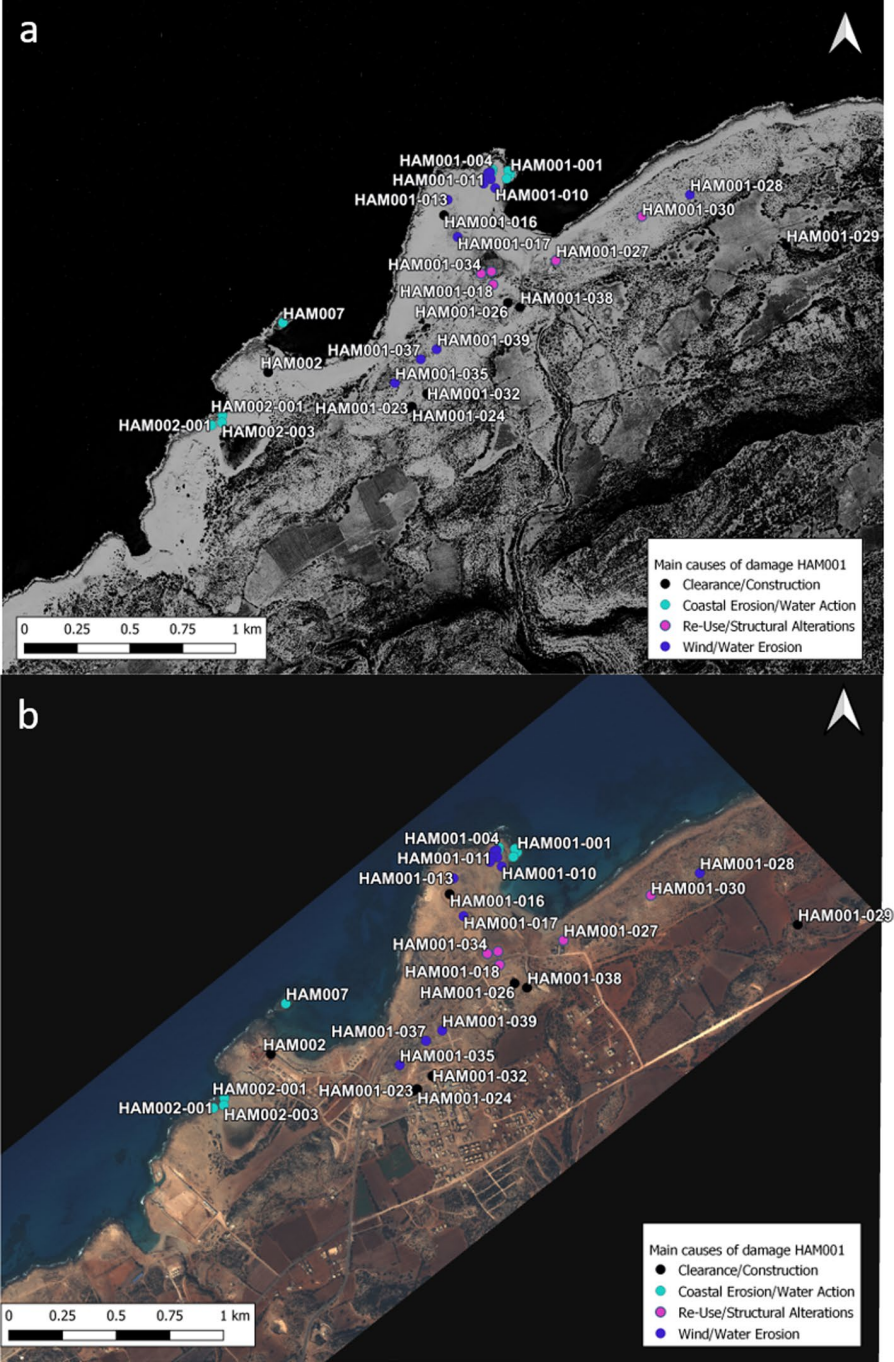
**a** Satellite image showing the area around HAM001 in 1966 when Zawiet El-Hamama only consisted of a few buildings and a small mosque. The shoreline below HAN007 is still intact (Basemap: declassified KH-7 satellite image, data available from the US Geological Survey). **b** The area around Phycus in November 2020, showing the location and main causes of damage to sub-sites. The shoreline below HAM007 has been significantly altered (Skysat Satellite Image, European Space Agency)

Phycus is located approximately 30 km (km) west of Apollonia on a small peninsula that extends out into the sea. It probably served as a secondary harbor to Cyrene and as the harbor of Balagrae, modern day Al-Bayda (Hesein 2015). While the site is not as large as the ports at Apollonia or Ptolemais, the dense archaeological remains suggest that it was once of considerable size (Hesein 2015). Phycus played an important role in the region’s economy, so much so that the port settlement was mentioned in ancient writings. For instance, in the early fifth century Synesius mentions that ships that anchored here transported cargo that was stored in Phycus’ warehouses to destinations such as Constantinople and Alexandria (Hesein 2015; Synesius, *Letters* 129; 133). The survey team visited 29 sub-sites at HAM001 including the headland where the port, warehouses, and the main settlement were located, as well as the hill south of the promontory that held cisterns, vats, kilns, buildings, and rock-cut tombs. HAM002 marks the western end of Phycus, including substantial walls, buildings, cisterns, and quarries. HAM007 is formed of two small rocky islets with quarries and perhaps basins extending out into the sea. These islets may have been connected to the land in antiquity (Fig. 4).

#### Clearance/Construction

**Fig. 5:**
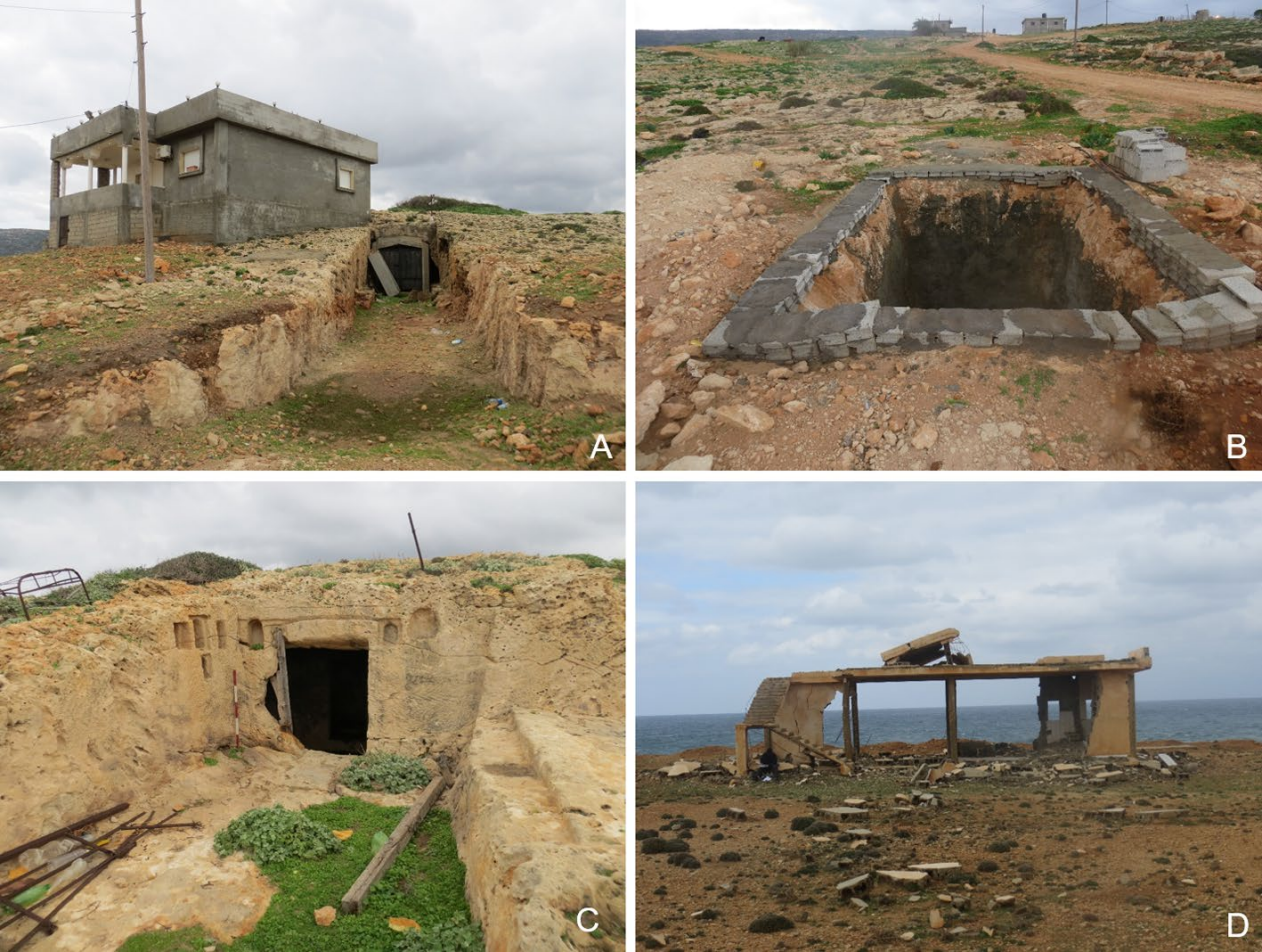
**a** At HAM001-026 a house was constructed on top of ancient buildings next to a quarry and very close to a rock-cut tomb. The rock-cut tomb is now blocked by a door and presumably used for storage. **b** A vat or cistern is relined and reused at HAM001-018. **c** Rock-cut tombs being repurposed as storage facilities and animal shelters at HAM001-039. **d** The now destroyed military building on top of HAM001-016. The area had been cleared by bulldozers before the military building was constructed

The modern settlement of Zawiet el-Hamama is located approximately half a kilometer south of the promontory of Phycus and has expanded considerably over the last decade. It developed from a small village of just a few houses in the 1960s to a more substantial settlement, which destroyed and damaged several archaeological features. The construction of several new access roads in 2014 indicates that Zawiet el-Hamama will continue to grow in the future. This action has already damaged two sites, HAM001-032 where foundation walls are visible and HAM001-039, which consists of a number of rectangular and round vats. This area was divided by the landowner in 2014 to be sold for profit (Hesein 2015). Here the main threat lies in the future developments of plots that were created by the new access roads, which will eventually be cleared for construction. Indeed, HAM001-023 and 024 have already been impacted by this process and are now destroyed by modern building development. Hesein (2015) recorded this set of buildings before the site was bulldozed, which was, at that point, already severely damaged. The remains of two further rectangular buildings (HAM001-026) were destroyed in 2015 when a house was built on the site (Fig. 5a).

Most of the clearance for development and construction is taking place in the immediate proximity of Zawiet el-Hamama. An exception is a military structure that was constructed in the center of the Phycus peninsula at some point before 2006 which has now fallen into disrepair (HAM001-016). In preparation for construction, the ground was completely bulldozed and most, if not all, of the archaeological features are now destroyed. Unfortunately, no archaeological record exists.

At HAM002 the large resort of El Shamariah now covers much of the western side of the bay. The clearance of the area for the construction of the resort most likely destroyed the features that were present here before they were recorded. Furthermore, illegal sand mining in the area around the resort was noted by Hesein in 2013, which destroyed any potential archaeological evidence (Hesein 2015). Indeed, comparing satellite imagery from 1966 to more recent images, it becomes clear that the shape of the beach has been significantly altered, probably as the result of sand mining and likely dredging, perhaps to create a more appealing sea front area for the resort (Fig. 4). Additional bulldozing and sand mining took place northeast of the resort from 2016 onwards, which is also clearly visible on satellite imagery. To the southwest of the resort three sub-sites of HAM002 still exist: a quarry (HAM002-003), the remains of a substantial wall, perhaps the enceinte of Phycus (HAM002-002), and a wall and ashlar blocks (HAM002-001). While these features do not show any obvious signs of damage by human intervention, their location at the seashore makes them vulnerable to erosion by wave action and inundation, particularly during winter storms.

#### R-use/Structural Alterations

Four sub-sites were recorded as being reused and structurally altered to various degrees. Most notably, some of the large rectangular vats at HAM001-018 and 033 were relined with cement and in the case of HAM001-033 covered with metal sheeting (Fig. 5b). At HAM001-018 a new well-head was constructed to reuse the old well. Rock-cut tombs at HAM001-026 and HAM001-030 are repurposed as storage facilities and animal shelters. The entrance is frequently closed off by a wooden door (Fig. 5a and c).

#### Looting/Dumping/Fire/Vandalism

Littering has been recorded across Phycus and plastic bottles, cans, and plastic bags can be found on many of the sub-sites. There has been no obvious evidence for looting at HAM001, although the relatively small amount of substantial building materials on the area of the ancient settlement suggests that stone may have been taken from the site for reuse elsewhere.

#### Coastal Erosion/Wind and Water Action

**Fig. 6:**
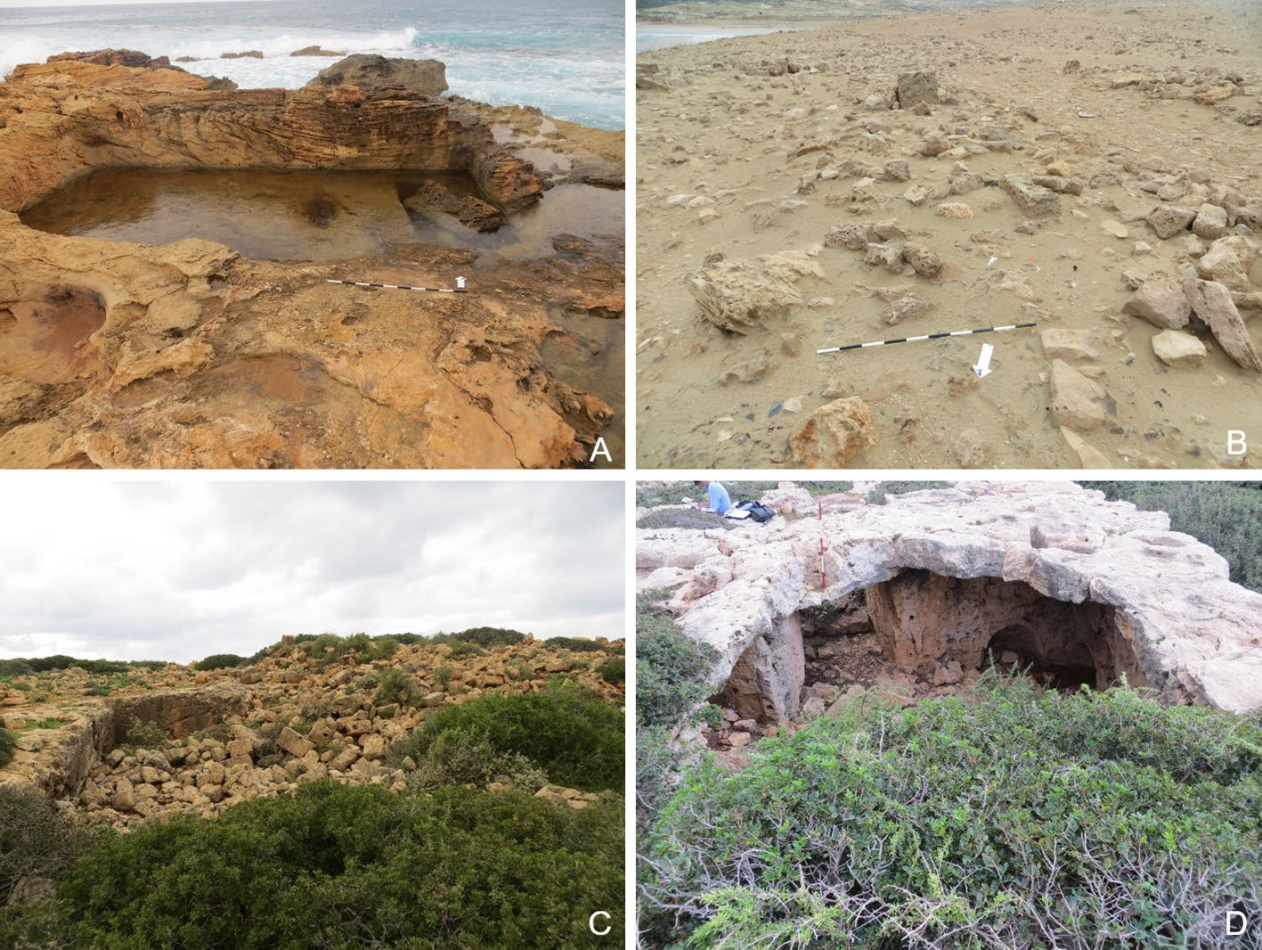
**a** The large rock-cut basin (HAM001-001) at the harbor of Phycus suffers from erosion caused by wave action and inundation. A part of the outer wall has fallen into the basin. **b** Wind and water action cause erosion across the Phycus promontory (HAM001-006). **c** Substantial building (HAM001-028) that has largely collapsed. Parts of the building are covered by low shrubs and bushes. **d** Collapsed cisterns at HAM001-028 showing signs of weathering and partly covered by vegetation

The sub-sites located on the promontory of Phycus are greatly affected by being exposed to the elements. The vats, basins, and warehouses at the port area of Phycus directly located by the seashore (HAM001-001 to 003) are impacted by waves which cause serious erosion and structural damage. This was noted by Jones and Little when they visited the site in the late 1960s. Comparing the photographs taken by Jones and Little (1971, Plate IV, 2) with the images from the CCS survey team show that the outer wall of a rock-cut tank that was still intact in the late 1960s has now collapsed into the tank, indicating this was caused by strong incoming waves and the increasing severity of winter storms (Fig. 6a).

HAM001-005 to HAM001-017 marks an area of ancient settlement and warehouses on higher ground, located well above the high-water mark. That this area was a busy part of ancient Phycus is evident from the amount of rubble and pottery that was strewn across the site. The remains have been exposed to the elements for a long time, eroded by wind and rain sweeping over the exposed promontory, resulting in many of the buildings being stripped down to their foundations (Fig. 6b). HAM001-017 is located on top of the low hilltop in the center of the promontory. This possible military fortlet was constructed of ashlar masonry supported by a revetment of smaller stones and rubble. Towers probably existed along the outer walls (Emrage 2015). While the site shows clear signs of erosion, much of its foundation and some of the lower courses of the walls are still standing. Sites located a little further inland also show signs of water and wind erosion, but they have not yet been stripped to their foundations (Fig. 6c).

#### Natural Vegetation

Only a few low shrubs and grasses grow on the Phycus promontory, and it is interesting to note that low bushes and grasses growing on sites further inland cause little damage to standing features and appear to provide a significant measure of protection (Fig. 6c and d).

### Aptouchos (HAN001)

**Fig. 7:**
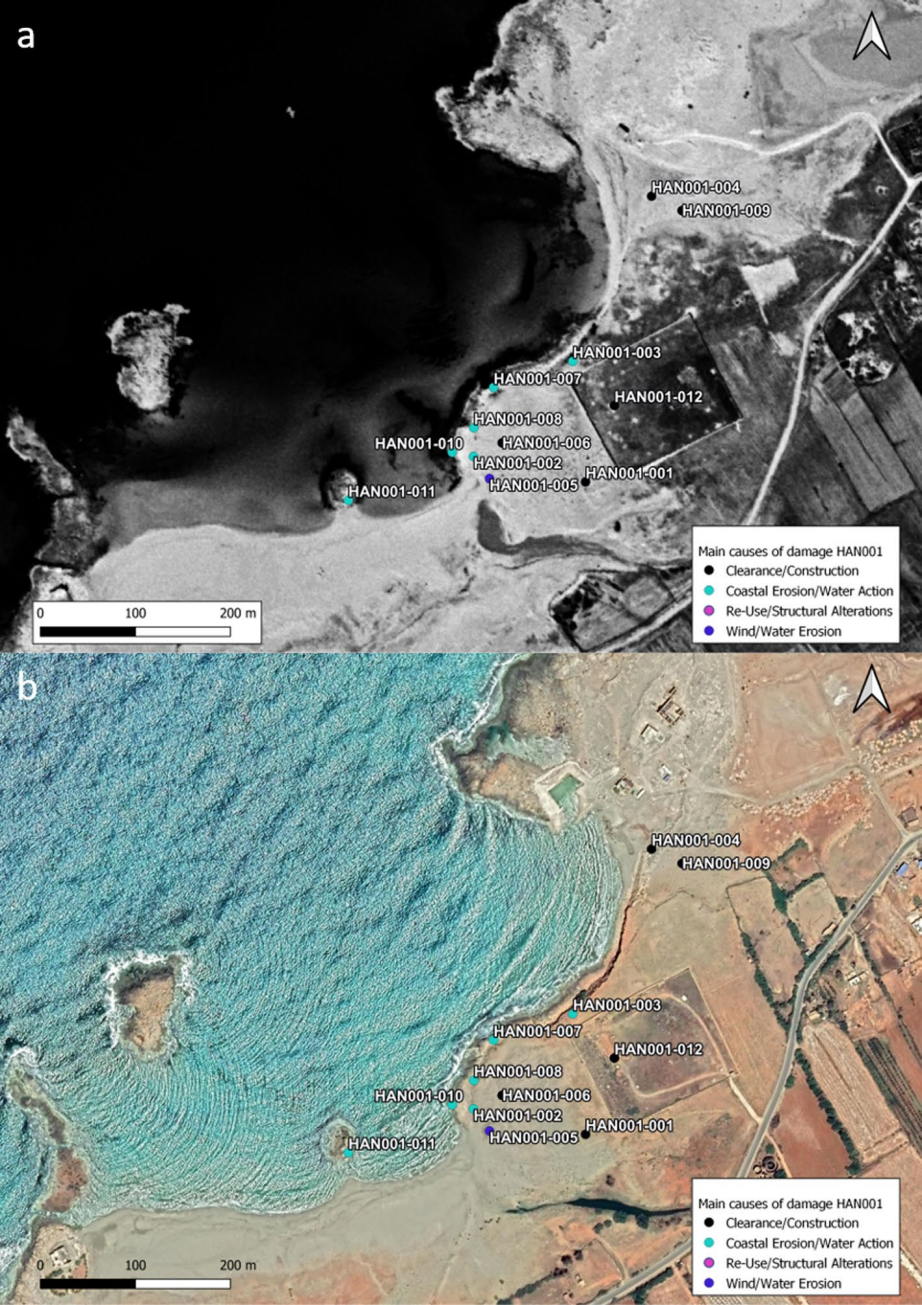
**a** Declassified KH-9 satellite image showing the area around HAN001 in 1975. The Islamic cemetery that is placed on top of the ancient settlement was already in existence (Basemap: declassified KH-9 satellite image, data available from the US Geological Survey). **b** Satellite image from 2019 showing the location of sub-sites and the causes of damage to them. The newly dug harbor basin is clearly visible at the northeastern edge of the bay (Basemap: ©Digital Globe via Google Earth)

Located approximately 14 km southwest of Phycus, Aptouchos consists of a settlement and harbor. Like Phycus the site occupies a large area, some of which is now covered by a large Islamic cemetery and the modern village of Zawiet el-Hanya (Fig. 7a and b). The small coves along the seashore provide a good location for harboring smaller ships (Jones and Little 1971). The area along the seashore contains evidence of industrial activity, including the remains of circular vats, tanks, and cisterns together with foundations of large buildings. A small rocky island (HAN001-011) holds a series of vats lined with *opus signum*. Small slots cut into the rock (0.2 m in diam.) suggest that the vats were perhaps covered by some sort of roof (Hesein 2014). The CCS survey team visited 13 sub-sites located along the shoreline and a small hill to the southeast.

#### Clearance/Construction

**Fig. 8:**
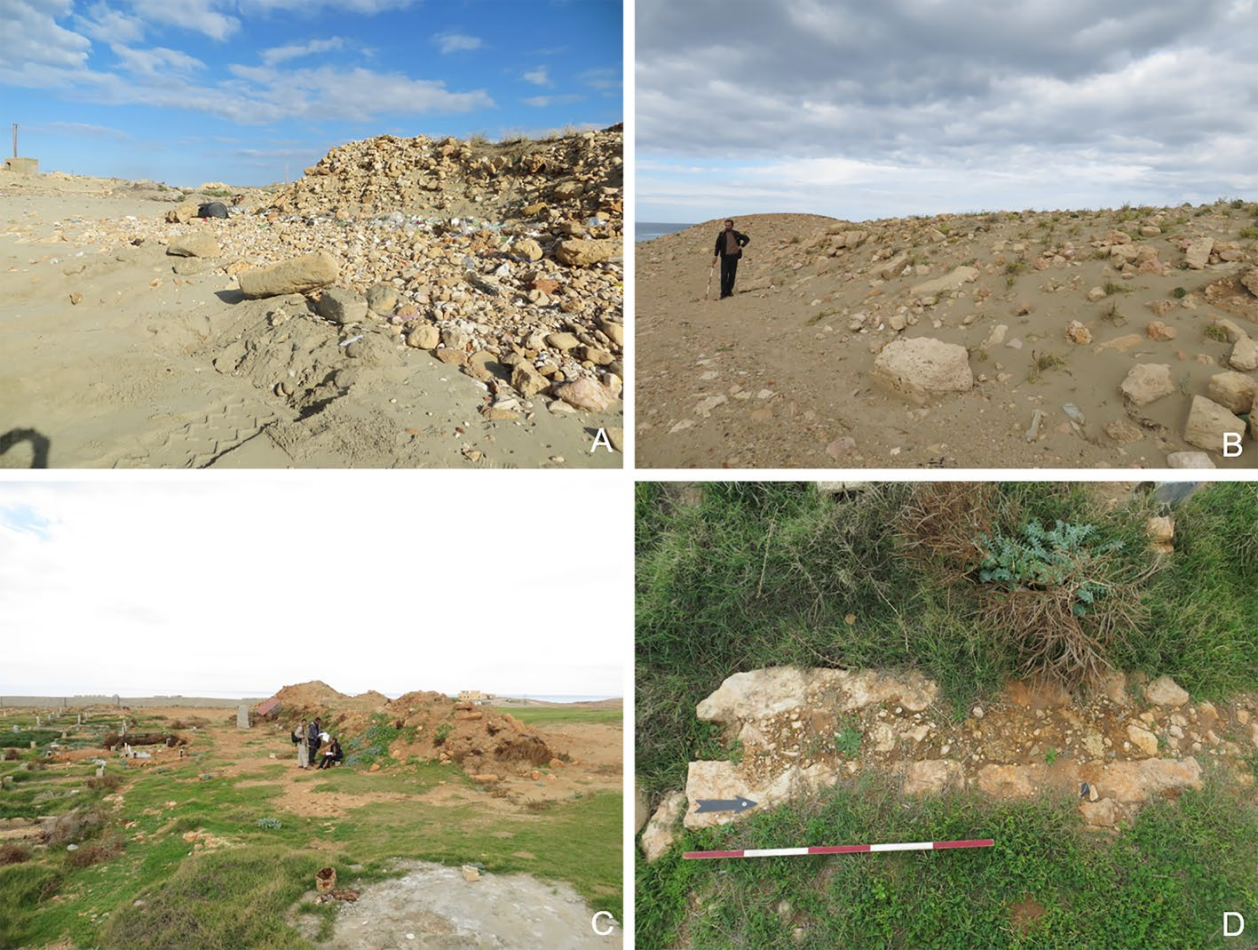
**a** Bulldozing and clearance tracks at HAN001-004. **b** Part of HAN001-005, showing bulldozed rubble and masonry from the hilltop site. **c** Clearance mounds from inside the Islamic cemetery at HAN001-012, containing rectangular blocks and rubble debris. **d** A small channel, perhaps relating to the former settlement of Aptouchos, is still visible within the Islamic cemetery

The modern village of Zawiet el-Hanya is located approximately 1 km southeast of the classical period site of Aptouchos. Today, a large Islamic cemetery covers most of the area where the ancient settlement was located. A large rectangular structure, mostly covered by sand, in the southwest corner of the Islamic cemetery has been damaged by bulldozing along its northeastern side (HAN001-001). Satellite imagery suggests that the bulldozing event happened between January and August 2014, when a small hill immediately to the northeast of HAN001-001 was landscaped (HAN001-006; Fig. 8b). Bulldozing tracks visible on the satellite image suggest the damage was caused by creating an access route to carry out the landscaping. KH-9 Hexagon satellite imagery from 1975 indicates that there may have been a rectangular building on top of the hill. Large amounts of pottery and dressed stone strewn across the area confirmed a high level of ancient activity. Since the clearance in 2014, no building or development activity has occurred. Clearance for the cemetery may have begun in 2013 and is ongoing. Recent earthworks were noted on the western side of the cemetery to create some space for new burials (HAN001-012; Fig. 8c and d). The presence of dressed building stones within substantial clearance mounds suggests that remnants of the former settlement present within the cemetery compound have now been destroyed.

In the northeastern part of the northernmost bay, the team recorded significant evidence for ground clearance and sand mining. A small hill to the north of the bay (HAN001-004; Fig. 8a) had been truncated by bulldozers to construct a military building in the recent past. Substantial bulldozing in this area had already occurred when Hesein visited the area in 2013, but large pottery scatters indicate significant activity in this area in antiquity (Hesein 2015). A further substantial change to the north bay occurred in 2019 when a small rectangular harbor basin was carved out of the rock. It is unclear how much archaeological evidence has been destroyed here, but satellite imagery shows rectangular vats or quarries in this area, so it is very likely that some features have vanished.

#### Looting/Dumping/Fire/Vandalism

Littering is evident across the site, particularly against the foreshore, together with some evidence of looting activity at the modern cemetery (HAN001-012) against the shoreline (HAN001-005), evidenced by small freshly dug pits.

#### Coastal Erosion/Wind and Water Action

**Fig. 9:**
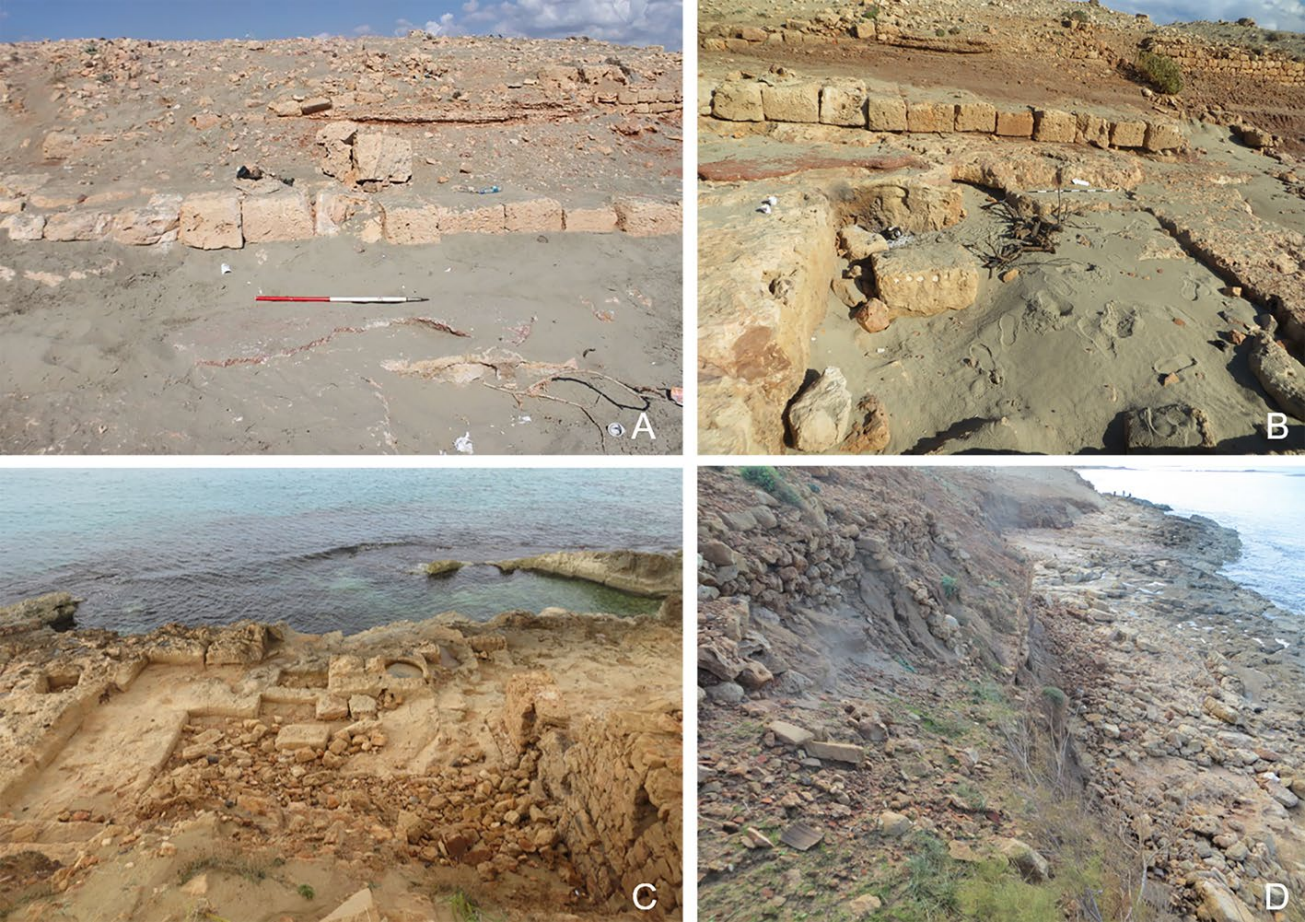
**a** Photograph taken by Hesein in 2011, the *opus signum* floor and the basin are both covered by sand (Hesein 2015, Figure 9–107). **b** The same area in 2020 is noticeably more exposed. The sand cover has been swept away exposing the *opus signum* floor and the basin. **c** At HAN001-007 erosion caused by incoming waves during severe weather leads to collapse and structural damage of still existing walls and features. **d** Severe erosion of the escarpment at HAN001-003 was caused by high energy waves during winter storms

Coastal erosion and deterioration caused by water and wind action is the second major cause of damage to the archaeological remains at Aptouchos. This is particularly apparent along the shoreline of the exposed escarpment of the northernmost bay (HAN001-003, Fig. 9d). The escarpment provides a near vertical section, exposing part of the ancient settlement, with multiple phases of buildings overlying bedrock. The loss of stratified deposits to the effects of winter storms is particularly severe and, in places, only rock-cut features now survive. A vaulted cistern that was protruding from the escarpment in the 1960s, documented by Jones and Little (1972), has now almost entirely disappeared. Other walls now protrude out of the eroding soil, and the beach below is littered with masonry from ancient buildings.

The level of erosion across the site is particularly striking when comparing images taken in 2011 by Hesein with photographs taken by the survey team nine years later. The photograph taken at HAN001-002 in 2011 shows the exposed wall of a building with at least three rooms and a series of vats lined with waterproof mortar, as well as the edge of *opus signum* floor covered by sand. Some erosion of the seaward escarpment can be seen in the background (Fig. 9a). By December 2020, when the CCS survey team visited the site, the *opus signum* floor was fully exposed to the elements and a vat in front of the back wall was uncovered. Raw signs of recent erosion can be spotted just behind the line of blocks that formed the wall, where dark red soil has been exposed (Fig. 9b). The impact of high energy wave action on the rock-cut features such as vats, basins, and quarries can be observed near the foreshore. Several features show signs of severe erosion, while others are being uncovered and exposed for the first time in millennia.

## Discussion

The survey area between Phycus and Al-Ogla provides a snapshot of the problems many coastal heritage sites face along the vast Libyan coastline that spans over almost 2000 km. As well as damage to sites, the survey team has also recorded potential threats. The threat assessment included issues that are currently ongoing and will continue to affect the site, as well as damage that may occur within the next five years based on past activity in and around the area. Our assessment predicts that activities surrounding clearance and construction (11 out of 14 sites) as well as agricultural activities (8 out of 14 sites) will become even more prevalent in the near future (Fig. 3).

The primary reasons behind the destruction and threat to these sites are closely linked to the country’s struggle with ongoing civil unrest and political instability over the last decade. Successive governments over the past sixty years showed little interest in the rich and unique archaeological heritage this country has to offer, resulting in insufficient support for the DoA who, in turn, struggled (and still struggles) to preserve and protect Libya’s cultural heritage (Abdulkariem and Bennett 2014; Kane 2015; Bennett 2018). The failure of the state to activate and enforce laws dealing with the protection of cultural heritage has emboldened developers, private builders, and farmers to bulldoze archaeological sites for the purpose of urban or agricultural expansion. Currently it is possible to carry out this illegal work without any consequences. Furthermore, the remote location of some sites, often on private land and property, has made them an ideal target for illegal excavations in a search for treasures. To protect Libya’s coastal cultural heritage for future generations to enjoy, it is indeed important for the new government of Libya to recognize its importance and to enforce legislation that is already in place (Law No. (3) of 1424 P.B (1994) for Protection of Antiquities, Museums, Old Cities and Historical Buildings). Without the government’s official support and upholding existing laws through policing and the courts of law, it is impossible for the DoA and other heritage professionals to carry out short-, medium-, and long-term mitigation and protection plans for the sites in the region. Collaboration with the Libyan government to identify measures that minimize the impact of erosion on archaeological sites is desired but proves difficult, as the country is still in the process of building a stable administration. At the time of writing, cultural heritage in general, and archaeological remains more specifically, are not yet a priority in government policy making and planning, and while the situation seems to improve steadily, much work is still needed to convince the government of the importance of heavily investing in the protection of cultural heritage across the country.

On a local level, raising awareness among the general public about the value of their own cultural heritage is of paramount concern (Abdulkariem 2013; Leone et al. 2020). With a rising population, housing is in short supply and, therefore, private individuals build houses where there is space, without permission or archaeological assessment of the area and, in the process, are destroying and damaging sites (Abdulkariem and Bennett 2014; Emrage 2015; Hesein 2015; Bennett 2018; Abdrbba 2019). The establishment of workshops and awareness raising campaigns is important, as it may prevent local landowners from destroying or selling their land when it is of archaeological value, or it might encourage them to return items of historical value they found on their land to the DoA, rather than breaking the law by selling them on the black market. The ‘Heritage for All’ campaign launched by the DoA in Cyrenaica in 2016 demonstrates the success of such programs, where Libyan heritage professionals organize events for the general public and school children to tell them about the value of protecting their own heritage. Success stories include, for instance, the return of funerary statues and pottery found in agricultural fields to the DoA (Anon. 2019a; Anon. 2019b). Furthermore, as the country’s political situation stabilizes, the integration of archaeological and rescue excavation during the planning process of new developments in the future will be vital to protect, or at the very least record, cultural heritage before it is destroyed.

**Fig. 10:**
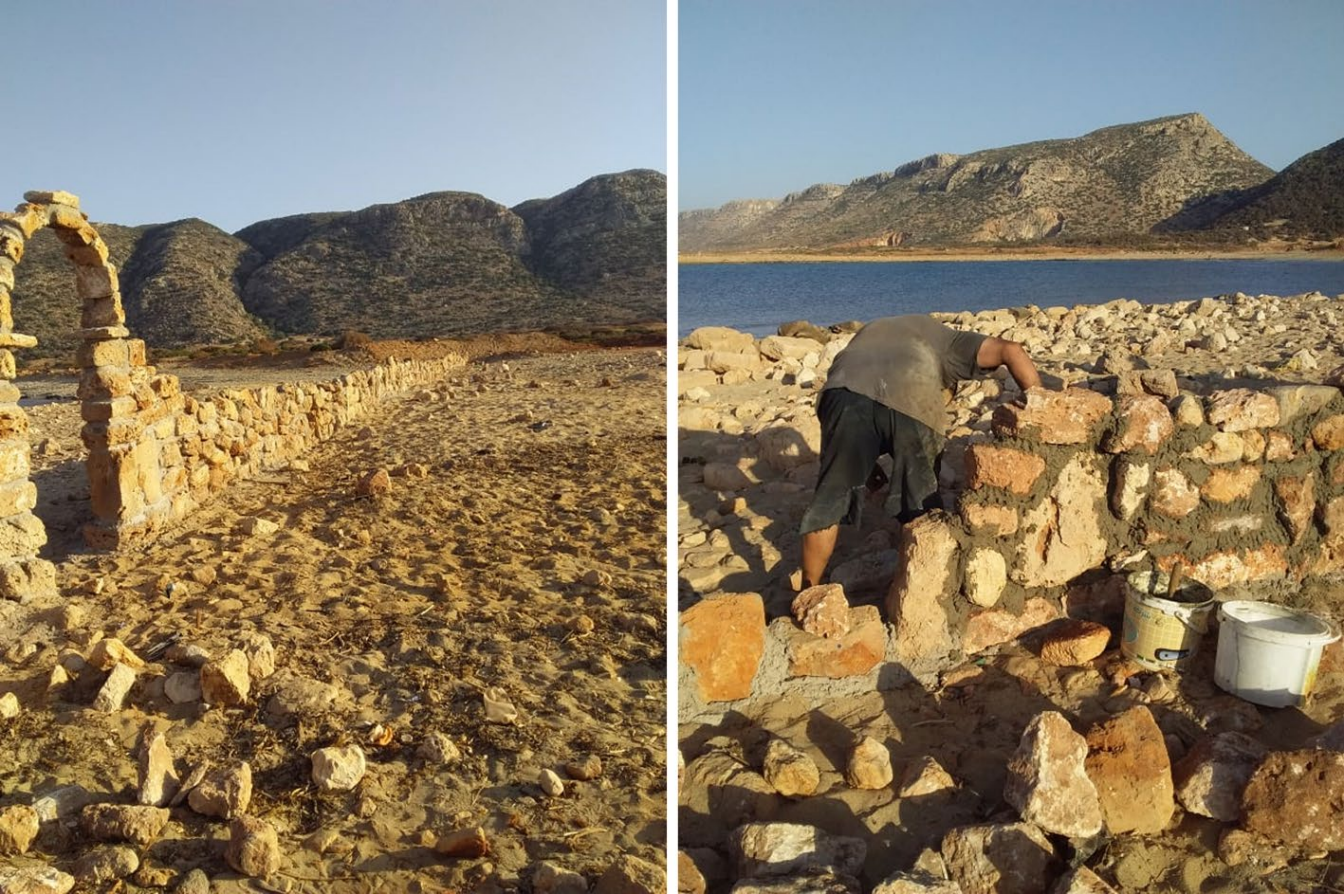
Perimeter wall constructed around Sidi Awlad Noah to mark the outline of the site to protect it from future development

Given the limited amount of intensive survey work undertaken at coastal sites, more work is needed to determine their size and extent to be able to erect protective fences and barriers where necessary. The production of information panels that can be placed on the sites can highlight their archaeological and historical importance to the public, and can also serve as a reminder that this property is protected by law. However, it is important to keep in mind that realistically, with the current speed of development along the Libyan coast, it will be impossible to save all the sites that the CCS survey has covered. Rescue excavations of imminently threatened heritage could, at the very least, ensure that a detailed record of the site exists. Financial support from foreign partners creates opportunities for such urgent and necessary interventions to protect archaeological sites. Some of the financial support received through the CCS survey enabled the construction of a perimeter wall around the coastal site of Awlad Sidi Noah after the second stage of the survey was concluded. Signage has been installed on the site detailing its importance and its protected status in law (Fig. 10).

The steady loss of these under documented coastal sites creates an ever-growing gap in our understanding of how local and Mediterranean-wide maritime networks developed, functioned, and changed over the centuries. Cyrenaica was ideally situated to connect the western and eastern Mediterranean, with its larger and smaller harbors playing important economic and political roles across the Mediterranean, while also connecting large inland cities such as Cyrene with the wider world. The many production sites documented during the survey, including farm estates, vats, basins, and kilns as well as dense pottery scatters, and pressing and grinding equipment, speak of an area that was heavily involved in the local and wider economies during the classical period. Hesein’s impressive study on the Cyrenaican Roman harbor sites and their typology only scratches the surface of the region’s importance within local and wider social, political and economic networks (Hesein 2015). Much more focused survey work and excavation are needed to understand the different functions of individual sites such as Phycus, Al Ogla or Aptouchos.

A positive development of recent years has been an increased effort by foreign missions to help with capacity building among Libyan heritage professionals to support the DoA in their efforts. In 2011, Bennett and Barker noted a ‘lack of skilled archaeological personnel on the ground to undertake the fieldwork and all the other aspects including reporting, conservation and curatorial care’ (2011, 23). Over the past five years, many Libyan archaeologists have received high level capacity-building courses in the fields of documentation, protection, and management of archaeological sites. These projects, which have been funded and directed by various international institutions, have contributed to develop and raise the capabilities of Libyan archaeologists, especially in the use of modern methodologies and techniques (Kane et al. 2017; Mugnai et al. 2017; Nikolaus et al. 2018, 2019; Hobson 2019; Leone et al. 2020). This is important as Libyan archaeologists increasingly gain the capacity and tools to slowly take control of the documentation and management of their own heritage, as well as the production of reports, site narratives, and databases.

MarEA provided training videos that cover documentation and survey methodologies specific to maritime archaeology and underwater recording during the duration of the CCS project. Additional materials covered the use of drones for documentation, the production of 3D imagery, and GIS mapping skills. Further training was provided in person led by the Libyan codirectors, including the use of the survey forms specific to damage and threat assessment, the use of handheld GPS, and how to use Google Earth to detect, monitor, and record archaeological sites.

The importance of cooperative projects such as the CCS also lies in the exchange of information resources between partners. Institutions from outside Libya may have access to satellite and other aerial and historical imagery that can support survey work on the ground and can contribute greatly to our knowledge of these sites, how they have changed over time, and what steps should be taken to protect them from various dangers that they are facing. Furthermore, continuous exchange of information and resources, transparent dialog and scientific discussions between Libyan archaeologists and foreign partners is enriching and insightful for everyone involved. Joint publications of scientific reports on the results of projects such the CCS contributes to this exchange; they raise awareness and may generate interest for potential future conservation and protection projects.

To further establish and progress the discipline of maritime archaeology, and the protection thereof, the CCS team has identified several needs which can provide the foundation for the development of a comprehensive framework for future in-country strategies:

### Training and Workshops

- Capacity development specifically related to maritime archaeology, including diving lessons for archaeologists interested in joining the diving team.
- Information sessions on current theory and methodology in maritime archaeology, including coastal, nearshore, and underwater components.
- In-person training in underwater recording techniques, including underwater photography and 3-D modelling.
- Training in GIS and remote sensing techniques to aid site monitoring and calculating past and future shoreline change.
- Workshops that specifically deal with maritime heritage conservation techniques.
- Opportunities for university students and members of the DoA to take part in local underwater training excavations and terrestrial coastal surveys. Here, knowledge gained from previous trainings that took place in Tunisia (Hobson 2019; Leone et al. 2020) could be passed on.

### Heritage Management and Protection Strategies

- Continue condition assessment surveys like the CCS on land and underwater to determine the threats and damages to heritage.
- Develop short-, medium- and long-term mitigation strategies to protect the sites.
- Contribute to developing guidance and policy documents that protect cultural heritage in the future.
- Further capacity building workshops specifically in the field of heritage management, monitoring, and protection, including training and workshops. These workshops should also involve members of law enforcement, including the Tourism Police and Coast Guard.
- Continue to develop outreach projects that raise awareness of the richness and importance of Libyan heritage from pre-history to more recent times.

### Fieldwork

- Carry out intensive terrestrial and diving surveys of the most threatened sites identified during the condition assessment survey.
- Carry out rescue excavations on sites that are immediately threatened by destruction.

### Equipment

- Equipment to carry out fieldwork underwater, such as diving gear, oxygen, and underwater recording equipment (e.g., camera or GoPro, recording grids).
- Relevant software to process data themselves.
- Powerful laptops/desktops to process data.

## Conclusion

The increase in human and natural impacts that threaten Cyrenaican maritime heritage, on both land and underwater, highlight the urgent need for the implementation of measures for their management and protection to preserve them for future generations. The ongoing coastal urban and agricultural development coupled with a rapidly increasing population, as well as the exacerbating effects of climate change, are all having a negative impact on the maritime cultural resource.

The data on the condition of maritime heritage derived from the CCS survey generate firsthand new knowledge that can be used to drive future strategies of research and heritage protection plans in Libya, including urgent mitigation and planning to tackle future challenges caused by our changing climate. In its collaboration with Libyan heritage professionals and authorities, the project is fostering the necessary maritime specific expertise among Libyan heritage professionals through training that focuses specifically on maritime archaeology. More broadly, the project has established grounds for strengthening and expanding the international network of partnerships in maritime cultural heritage and in raising awareness of endangered maritime archaeology in Libya.

Ultimately, this project demonstrates the mechanism and requirements for a successful remote collaboration between UK institutions, in-country collaborators, and heritage authorities. This involves the sharing of research, resources, responsibilities, expertise, and building capacity to guarantee a successful completion of project objectives. The COVID-19 pandemic has exacerbated the need in heritage initiatives to adopt research frameworks that build on such strong networks. Travel restrictions have severely hampered international fieldwork. Consequently, there is a need to embrace alternative and long lasting approaches that build on the sharing of expertise between remote researchers and field teams. This is particularly important to archaeological research in the MENA region which, in its legacy, has relied on the presence and skills of international teams managing and undertaking fieldwork. Given growing urban development and littoralization in the MENA region, and impacts induced by environmental and climatic cycles, such datasets are valuable in determining the condition of the maritime resource and mitigating against these natural and anthropogenic pressures. Developing a collaborative heritage documentation and management strategy, alongside Libyan partners, can ultimately constitute a replicable approach that can be implemented in other coastal areas of Libya and across the MENA region.

## Data Availability

Available on request.
